# Salutatory

**Published:** 1874-11

**Authors:** W. F. Westmoreland


### Editorial.

---

SALUTATORY.—Eighteen months ago I fell a victim to disease, the result of a poisoned wound, and, after months of suffering and a tardy recovery, severed my connection with the JOURNAL. I do not propose here to discuss the ability and zeal with which the JOURNAL has been conducted for the past year, as that is patent to all its readers, but simply to announce that my health is completely restored, and that with this number I resume my former connection with it.

W. F. WESTMORELAND, M.D.

